# Dietary intake, lung function and airway inflammation in Mexico City school children exposed to air pollutants

**DOI:** 10.1186/1465-9921-10-122

**Published:** 2009-12-10

**Authors:** Isabelle Romieu, Albino Barraza-Villarreal, Consuelo Escamilla-Núñez, Jose L Texcalac-Sangrador, Leticia Hernandez-Cadena, David Díaz-Sánchez, Jordi De Batlle, Blanca E Del Rio-Navarro

**Affiliations:** 1 Instituto Nacional de Salud Pública, Cuernavaca, Mexico; 2 Human Studies Division, United States Environmental Protection Agency, Chapel Hill, North Carolina, USA; 3 Centre for Research in Environmental Epidemiology (CREAL), Barcelona, Spain; 4 CIBER Epidemiología y Salud Pública (CIBERESP), Barcelona, Spain; 5 Hospital Infantil de México Federico Gómez, Mexico, DF, Mexico

## Abstract

**Introduction:**

Air pollutant exposure has been associated with an increase in inflammatory markers and a decline in lung function in asthmatic children. Several studies suggest that dietary intake of fruits and vegetables might modify the adverse effect of air pollutants.

**Methods:**

A total of 158 asthmatic children recruited at the Children's Hospital of Mexico and 50 non-asthmatic children were followed for 22 weeks. Pulmonary function was measured and nasal lavage collected and analyzed every 2 weeks. Dietary intake was evaluated using a 108-item food frequency questionnaire and a fruit and vegetable index (FVI) and a Mediterranean diet index (MDI) were constructed. The impact of these indices on lung function and interleukin-8 (IL-8) and their interaction with air pollutants were determined using mixed regression models with random intercept and random slope.

**Results:**

FVI was inversely related to IL-8 levels in nasal lavage (p < 0.02) with a significant inverse trend (test for trend p < 0.001), MDI was positively related to lung function (p < 0.05), and children in the highest category of MDI had a higher FEV_1 _(test for trend p < 0.12) and FVC (test for trend p < 0.06) than children in the lowest category. A significant interaction was observed between FVI and ozone for FEV_1 _and FVC as was with MDI and ozone for FVC. No effect of diet was observed among healthy children.

**Conclusion:**

Our results suggest that fruit and vegetable intake and close adherence to the Mediterranean diet have a beneficial effect on inflammatory response and lung function in asthmatic children living in Mexico City.

## Introduction

Exposure to air pollution has been associated with decrements in lung function [1-3] and an increase in respiratory symptoms [4], effects to which asthmatic children appear more susceptible [5,6]. We have recently shown that exposure to fine particles (PM_2.5_) and ozone (O_3_) results in acute airway inflammation and a decrease in lung function in both asthmatic and non-asthmatic children [7]. In a randomized controlled trial among asthmatic children, we also showed that antioxidant supplementation (vitamin C and E) modulates the adverse effect of O_3 _on lung function and inflammatory response [8]. Fruits and vegetables are rich in antioxidants, in particular vitamin C and carotenoids, and higher intake has been related to better lung function in both adults [9] and children [10]. The Mediterranean diet has been shown to have a high antioxidant potential [11] and a beneficial effect on the risk of rhinitis [12] and asthma [13] in children and on lung function in adults[9]. In this study, we evaluated the impact and the potential modulating effect of dietary intake on lung function and airway inflammation among Mexico City school children exposed to high levels of air pollutants.

## Materials and methods

### Study design

A dynamic panel (cohort) study of 6 to 14 year-old asthmatic and non-asthmatic school children living in Mexico City was conducted between June 2003 and June 2005. The asthmatic children (n = 158) were patients of the Federico Gomez Hospital Infantil de Mexico. The severity of diagnosis of their asthma was based on clinical symptoms and response to treatment and rated by a pediatric allergist as mild (intermittent or persistent), moderate or severe according to Global Initiative for Asthma (GINA) guidelines [14]. Fifty non-asthmatic children were recruited by asking the asthmatic children to invite a schoolmate or a friend from their neighborhood; dropped out early after recruitment (9%). The children were enrolled during the first 10 months of the study (June 2003 - April 2004) and followed for an average of 22 weeks. Spirometric tests, measurements of interleukin-8 (IL-8) levels in nasal lavage and anthropometry measurements were conducted every 2 weeks during follow-up. The study methodology has been described elsewhere [7].

All procedures were explained to the parents, who signed an informed consent form. The children also gave their informed consent. The study protocol was reviewed and approved by the ethics committees at both the National Institute of Public Health and the Hospital Infantil de Mexico.

We collected data on sociodemographic variables including mother and father education, the type of school attended by the child, past health history and potential indoor environmental exposures. Allergy test results and information on medication and medical visits over the previous 2 years were obtained from the medical record.

### Spirometry

The spirometric tests were performed according to American Thoracic Society (ATS) specifications [15] using an EasyOne spirometer providing age, gender and height standardized pulmonary functions (ndd Medical Technologies, Andover, MA, USA). All lung function tests were performed by the same technicians, and the best of three technically acceptable tests was selected. Prior to the spirometric test, children answered a questionnaire on respiratory symptoms and had a clinical exam. If a child was diagnosed with respiratory infections, the spirometry was not done that day and was rescheduled for another day.

### Nasal lavage

Nasal lavage was performed following the methodology proposed by Diaz-Sanchez et al. [16], with the children sitting with the nasopharynx closed while tilting their neck back 45 from vertical. Five ml of warm (37°C) normal saline is instilled into each nostril by pipette. After 10 seconds, during which the subject shakes their head softly from side to side, they bring their head forward, expelling the wash fluid into a plastic receptable. The subject then performs up to four further nasal washes at 30-second intervals, with each wash being collected in a separate tube. We measured different cytokines including IL8, interferon gamma, IL6 and IL10 levels in nasal lavage in the laboratory of Dr. Diaz-Sanchez, using commercially available Elisa kits according to the manufacturer's instructions. However, except for IL8, the levels in most of the samples were under the detection limit and we report only the IL8 results. For logistic reasons, we did not determine cellular composition.

### Exhaled Nitric Oxide Levels

The levels of FeNO were measured following the ATS guidelines [17] during outpatient visits to a clinic. Children were seated for at least 5 min before commencing the measurement and throughout the procedure; all measurements were conducted indoors to minimize external inhaled NO-free external air. NO was measured by chemiluminescence, using a continuous analyzer (CDL 88 sq Michigan, USA). The FeNO reading was displayed in the monitoring system and the mean of the three acceptable tests was taken.

### Exhaled Breath Condensate Collection

EBC was collected using an R-tube and the breath was cooled by placing an aluminum cooling sleeve over the disposable polypropylene tube [18]. Samples were obtained following the ATS/ERS Task Force recommendations [18,19]. Participants were asked to breathe tidally through the mouthpiece connected to the R-tube for 10 minutes to collect approx. 2 ml of exhaled breath fluid, which was aliquoted and frozen to -70 C within 15 minutes of collection.

### Dietary assessment

At baseline, the mothers completed a 108-item food frequency questionnaire. A commonly used unit or portion size was specified for each food item (slice, glass, or natural unit such as one apple) and each mother was asked how often, on average, her child had consumed that amount over the previous year. Eight possible responses were given: 4 or more times per day; 2-3 times per day; once per day; 5-6 times per week; 2-4 times per week; once per week; 1-3 times per month, and; never or less than once per month [20]. We assigned proportional weight to each frequency of consumption in order to obtain a daily intake for all items provided in the food frequency questionnaire. The following weights were assigned: never or less than once per month = 0, 1-3 times per month = 2/30, once per week = 1/7, 2-4 times per week = 3/7, 5-6 times per week = 5.5/7, once per day = 1, 2-3 times per day = 2.5 and 4 or more times per day = 4.

The questionnaire was adapted to the Mexican population from the one developed by Willett *et al. *and validated in this population [20-22]. This dietary information was used to calculate the daily consumption of 8 food groups: fruits, vegetables, cereals, legumes, dairy products, meat, fish and junk food.

From these groups, two food consumption indices were constructed: a fruit and vegetable index (FVI) and a Mediterranean diet index (MDI). 1) The FVI was based on the intake of fruits and vegetables and the consumption of vitamins or supplements reported on the questionnaire and were scored on a scale of 0 to 3. Children whose consumption was below the median value were assigned 0, while children whose consumption was at or above of the median value were assigned 1. Additionally, the score was increased by 1 if the child reported consumption of vitamin C or supplement. This index was used as a continuous variable and a categorical variable considering 4 groups (0,1,2,3). 2) The MDI was adapted from that designed by Trichopoulou [23] and was generated from the sum of the 8 food groups to evaluate adherence to the Mediterranean dietary pattern. This index was generated as follows: i) The moderate alcohol consumptions component was suppressed as it was not applicable to children: ii) The high monounsaturated/saturated consumptions were eliminated because fat ratio component as it could not be computed from the available data: iii) And a junk food component was introduced as a previous study had suggested including fast food, snacks and sweets in Mediterranean diet scores [24]. In the case of fruits, vegetables, cereals, legumes, dairy products and fish, a value of 1 was assigned if consumption was above of the median value and 0 otherwise. For meat and junk food, the scoring was reversed. The scores for each food group were then summed to obtain a total score ranging from 1 (minimum adherence) to 4 (maximum adherence). This score was used a continuous variable and a categorical variable regrouping the score in four categories (category 1 = score 1 to 3, category 2 = score 2, category 3 = score 3, category 4 = score 6 to 8).

### Air pollutant exposure assessment

Exposure was estimated from the concentrations of outdoor fine particles (PM_2.5_), nitrogen dioxide (NO_2_) and ozone (O_3_) recorded by the Mexico City government at four fixed-site central monitoring (RAMA) locations in the study area. Daily average, maximum moving average and 8-hour maximum concentrations and meteorological data (temperature and humidity) were obtained for all 505 days of the study period. The home of each child was geo-referenced using a geographic information system and the closest monitoring station was assigned to the child. All children attended public schools located close to their home and no fixed-site monitoring station was more than 5 km from a child's home or school.

### Statistical analysis

The basic characteristics of the two groups of children were compared by bivariate analysis using the t-test, the Fisher exact test or the *χ*^2 ^test, depending on variable type. The associations between diet indices and health outcomes were evaluated using linear mixed effects models with random intercept, considering models for continuous response. These models account for repeated measurements in the same individuals enabled us to appreciate the variability within and between subjects.

The model is as follows:(1)

Where; i: represents the observation in the subject i. Y_j_, corresponds to the dependent variables, X_i _are the independent variables with fixed effects and *ε*_i _vector of residual components. A further advantage of the models used is that they do not discard subjects with incomplete data and take into account the correlation among repeated measurements in the same individual. The goodness of fit of each model was determined using residual diagnosis and the Hausman specification test [25]. Data on O_3_, PM_2.5 _and NO_2 _was included in our regression models on the basis of a previous analysis of these pollutants [7], which are known to affect pulmonary function and inflammatory markers. Models were adjusted for potential confounding, including gender, body mass index, previous day minimum temperature, corticoid use and chronological time. Other variables such as age, socioeconomic index (based on maternal education and school type), outdoor activities, atopic status, exposure to environmental tobacco smoke, use of anti-allergy medicine and season were not significant (p > 0.10) and did not alter the results by >1%. We also tested for interaction between air pollutant exposure and dietary intake to assess any modifying effect of diet on the adverse effect of air pollution on lung health including interaction term in our models and also evaluating the effect of our nutritional indices in children exposed to low and high levels of pollutants. We calculated the percent of change in IL-8 and pulmonary function in relation with FVI and MDI using the coefficients from our regression models divided by the corresponding baseline characteristics. Analyses were conducted using STATA 9.2 (Stata Corp., College Station, Tx, USA).

## Results

Table 1 presents the characteristics of the study population. The median age of participants was 9.6 years (Q25:7.9, Q75:11.0) for the asthmatic children and 9.3 (Q25:7.9, Q75:11.5) for the non-asthmatic children. Fifty-five percent of the asthmatic children were classified as having mild intermittent, 26.9% as having mild persistent and 17.5% as having moderate persistent asthma according to GINA guidelines. Eighty-nine percent of the asthmatic children and 72% of the non-asthmatic children had positive skin prick tests. The most common allergens sensitivities appear in the table 1.

**Table 1:** Baseline characteristics of the study population: 158 asthmatic and 50 non-asthmatic children living in Mexico City, 2003-2005

	Asthmatic (n = 158)		Non-asthmatic (n = 50)	
	
Characteristics	Median^§ ^(Q25, Q75)		Median (Q25, Q75)	
Sex, % masculine	61.9		40.0	
Age, years	9.6	(7.9, 11.0)	9.3	(7.9, 11.5)
Weight, kg	36.0	(27.0, 46.0)	32.0	(26.0, 45.0)
Height, cm	137.0	(124.5, 147.0)	134.0	(127.0, 147.0)
BMI, Kg/m^2^	18.9	(16.3, 22.0)	18.1	(15.7, 21.5)
Maternal education, years (mean (SD))	9.8	(3.0)	9.3	(3.0)
*Smoking at home, %*				
Father	54.8		45.0	
Mother	41.1		28.6	
Other person	23.4		29.1	
*Allergy prink test positive *(Atopics) (%)	88.8		80.0	
*Main allergens testing (%)*				
Cat	28.1		27.3	
Dog	20.0		21.8	
House dust mite	61.9		45.4	
Cockroach	41.9		30.9	
*Asthma diagnosis, %*				
Moderate persistent	17.5			
Mild persistent	26.9			
Mild intermittent	55.0			
*Baseline lung function and IL-8 levels*				
FEV_1_, L (mean (SD))	1.9	(0.7)	1.95	(0.6)
FVC, L (mean (SD))	2.3	(0.8)	2.25	(0.7)
IL-8, pg/ml^§^	157.2	(78.2, 295.1)	202.9	(112.9, 333.6)

SD: Standard deviation

^§ ^Mann-Whitney test [median (Q25, Q75)]

### Dietary intake

Table 2 presents the daily dietary intake of the children by food group and frequencies. There was no significant difference between the asthmatic and the nonasthmatic children. We observed a high frequency of intake of fruit or fruit juices, vegetables and dairy products as well as junk food. The most frequently consumed fruits were oranges, mandarins, apples, grapes, mangoes and grapefruits. Sixty nine percent of the asthmatic children and 70.9% of the non-asthmatic children were reported as consuming two or more vegetables per day. The most frequently consumed vegetables were tomatoes, zucchinis and chili peppers. The intake of junk food was also high in both groups, while the intake of fish was low. A total of 64.8% of the asthmatic children and 76.4% of the non-asthmatic children were reported as consuming two or more junk foods per day. Vitamin supplementation (mostly vitamin C) was high in both groups. No difference in diet index (FVI or MDI) scores was observed between the two groups.

**Table 2:** Dietary intake frequency for specifics food group and diet index scores among 208 children living in Mexico City, 2003-2005

Characteristics	Asthmatic (n = 158)	Non-asthmatic (n = 50)
*Fruit or fruit juice *(%)		
At least 4 times a month	4.4	1.8
2-6 times per week	34.2	23.6
More than once per day	61.4	74.6
*Vegetables *(%)		
At least 4 times a month	1.9	1.8
2-6 times per week	28.3	27.3
More than once per day	69.8	70.9
*Cereals or grains *(%)		
At least 4 times a month	0.6	0.0
2-6 times per week	21.4	20.0
More than once per day	78.0	80.0
*Legumes *(%)		
At least 4 times a month	28.9	38.2
2-6 times per week	50.9	50.9
More than once per day	20.1	10.9
*Dairy products *(%)		
At least 4 times a month	1.3	0.0
2-6 times per week	8.8	12.7
More than once per day	89.9	87.3
*Meat*		
At least 4 times a month (%)	6.9	10.9
2-6 times per week (%)	76.1	76.4
More than once per day (%)	17.0	12.7
*Fish *(%)		
At least 4 times a month	76.7	74.6
2-6 times per week	22.6	23.6
More than once per day	0.6	1.8
*Junk food *(%)		
At least 4 times a month	0.6	1.8
2-6 times per week	34.6	21.8
More than once per day	64.8	76.4
*Calories, kcal*		
(median (Q25, Q75))^§^	1797.4(1594.9, 2194.4)	1795.2(1569.6, 2311.9)
*Vitamins *(%)	54.4	56.4
*Fruit and vegetable index *(%)		
0	14.4	14.5
1	33.1	25.4
2	36.2	43.6
3	16.2	16.4
*Mediterranean diet index *(%)		
1	37.5	41.8
2	18.1	25.4
3	23.7	16.4
4	20.6	16.4

^§ ^Mann-Whitney test [median (Q25, Q75)]

### Air pollution exposure data

The 8-hour moving average PM_2.5 _ranged from 4.24 to 102.8 μg/m^3 ^during the study period, with a mean of 28.9 μg/m^3^. It exceeded 30 μg/m^3 ^on 52% of the days. The 8-hour moving average of NO_2 _ranged from 14.9 to 77.6 ppb, with a mean of 37.4 ppb. The 8-hour moving average of O_3 _ranged from 4.9 to 86.3 ppb, with a mean of 31.6 ppb (Table 2). The correlation between PM_2.5 _and O_3 _was r = 0.46 (p = 0.000). The correlations between O_3 _and NO_2 _and NO_2 _and PM_2.5 _were r = 0.28 (p = 0.000) and r = 0.61 (p = 0.000), respectively. Local measurements conducted at the children's schools were correlated with concentrations at the central monitoring stations (r = 0.77 for PM_2.5_, r = 0.21 for NO_2 _and r = 0.60 for O_3_). Mean local measurements were 26.3 μg/m^3 ^(standard deviation (SD) = 12.5) for PM_2.5_, 35.05 ppb (SD = 12.6) for NO_2 _and 26.9 ppb (SD = 9.5) for O_3_.

### Association of FVI with lung function and IL-8

The associations between FVI and main outcomes for the asthmatic and nonasthmatic children are shown in Table 3. After accounting for air pollutants (ozone and PM_2.5_) and confounding factors, FVI was significantly related to lower IL-8 and positively related to lung function. For each 1-unit increase in FVI there was a significant decrease in IL-8. We calculate that children in the highest intake level of the FVI index (4) had 8% lower IL8 than children with the lowest intake. FVI was not significantly related to exhaled NO but we observed a positive association with exhaled breath PH, although non-significant.

**Table 3:** Association of inflammatory markers and lung function in school children living in Mexico City with exposure to Fruit and vegetable index and Mediterranean diet index, 2003-2005

	Fruit and vegetable index			Mediterranean diet index		
		
Models	Coefficient* (SE)	P value	P value^&^	Coefficient* (SE)	P value	P value^&^
	**Asthmatic**					
*Inflammatory markers *^†^						
ln IL-8	-0.136(0.055)	0.013		-0.029(0.032)	0.358	
lnFeNO	-0.021(0.086)	0.812		-0.023(0.050)	0.649	
pH	0.035(0.026)	0.179		-0.009(0.015)	0.546	
*Lung function *^‡^						
FEV_1 _(L)	0.074(0.053)	0.165	0.023	0.058(0.029)	0.045	
FVC (L)	0.105(0.058)	0.073	0.008	0.075(0.032)	0.018	0.020
FEF25-75 (L)	0.048(0.078)	0.539		0.050(0.043)	0.241	
	**Non-asthmatic**					
*Inflammatory markers *^†^						
ln IL-8	-0.133(0.107)	0.214		0.063(0.063)	0.312	
lnFeNO	0.373(0.178)	0.036		0.154(0.115)	0.181	
pH	0.041(0.052)	0.434		-0.015(0.030)	0.623	
*Lung function *^‡^						
FEV_1 _(L)	-0.030(0.074)	0.689		-0.016(0.047)	0.730	
FVC (L)	0.005(0.081)	0.952		-0.025(0.052)	0.622	
FEF25-75 (L)	-0.131(0.129)	0.309		0.047(0.080)	0.557	

* Coefficients correspond to a change in the ln IL-8, LnFeno, pH or lung function for a change in one unit of the FVI or MDI.

^† ^Models for inflammatory markers included 753 inflammatory marker measurements for 119 asthmatic children. Models are adjusted for the following variables: same day exposure: 24-hr average O_3 _(pbb), 24-hr average PM_2.5 _(μg/m^3^), previous day minimum temperature, gender, body mass index, calories, corticoid use and chronological time.

^‡ ^Models for lung function included 1503 lung function measurements for 158 asthmatic children.

Models are adjusted for the following variables: 5-day accumulated moving average O_3 _(ppb), 5-day accumulated average (maximum) PM_2.5 _(μg/m^3^), previous day minimum temperature, gender, body mass index, calories and chronological time.

^&^p value of interaction between ozone and indices (FVI or MDI)

FVI was also positively related to forced expiratory volume in one second (FEV_1_) and forced vital capacity (FVC). The effect was marginally significant for FVC. A 1-point increase in FVI was associated with a 105 ml (nearly 5%) increase in FVC.

**Figure 1:**
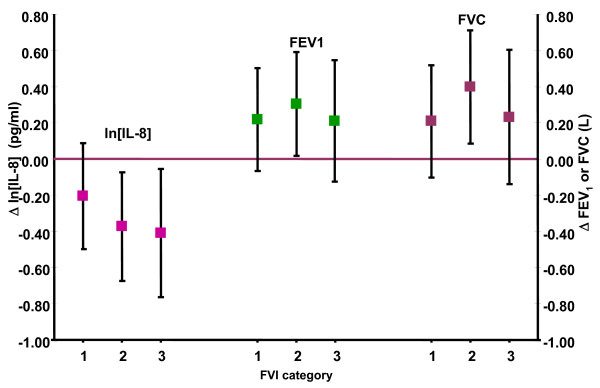
**Association between ln [IL-8] and lung function in asthmatic children living in Mexico City by categories of fruit and vegetable index (FVI) 2003-2005**. [ln IL-8] model was adjusted for gender, body mass index, calories, 24-hr average O3 (pbb), previous day minimum temperature, corticoid use and chronological time. Lung function models were adjusted for gender, body mass index, calories, 5-day accumulated moving average O3 (ppb), previous day minimum temperature and chronological time. Reference category 0.

When FVI was analyzed as a categorical variable, we observed a significant decreasing trend in IL-8 levels with increasing categories of FVI (p = 0.001) but no clear trend was observed for lung function (Figure 1).

**Figure 2:**
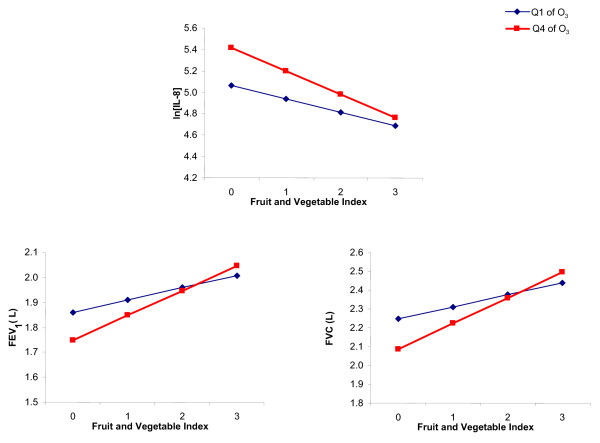
**Interaction between fruits and vegetables index and ozone levels (Q1-Q4) for IL8, FEV_1 _and FVC**. Q1 = ≤ 25 ppb, max 8-h moving average Q4 = ≥ 38 ppb, max 8-h moving average.

We observed a significant positive interaction between ozone level and FVI for both FEV_1 _(p = 0.023) and FVC (p = 0.008) suggesting that the protective effect of FVI increased with higher ozone levels (Figure 2). When data were stratified by low (≤ 25 ppb, max 8-h moving average) and high (≥ 38 ppb, max 8-h moving average) ozone levels, we observed that the positive effect of FVI was significantly larger when exposure to ozone was in the highest quartile. (Table 4)

**Table 4:** Association of inflammatory markers and lung function with exposure to Fruit and vegetable index and Mediterranean diet index stratified by the highest and lowest concentrations of ozone in school children living in Mexico City, 2003-2005

	*First quartile of O*_3 _*(ppb)*		*Fourth quartile of O*_3 _*(ppb)*	
		
	Coefficient*(SE)	P value	Coefficient*(SE)	P value
Models with exposure to FVI				
Ln IL-8^†^	-0.125(0.094)	0.182	-0.219(0.084)	0.009
FEV_1 _^‡ ^(L)	0.049(0.061)	0.415	0.099(0.058)	0.089
FVC^‡ ^(L)	0.065(0.069)	0.346	0.137(0.066)	0.037
				
Models with exposure to MDI				
Ln IL-8^†^	-0.020(0.055)	0.723	-0.022(0.048)	0.627
FEV_1 _^‡ ^(L)	0.048(0.033)	0.149	0.051(0.032)	0.113
FVC^‡ ^(L)	0.048(0.037)	0.203	0.081(0.036)	0.023

* Coefficients correspond to a change in the ln IL-8 or lung function for a change in one unit of the FVI or MDI.

^† ^[ln IL-8] model included the following variables: same day exposure: 24-hr average O_3 _(pbb), gender, body mass index, calories, previous day minimum temperature, corticoid use and chronological time.

^‡ ^Models for lung function included the following variables: 5-day accumulated moving average O_3 _(ppb), gender, body mass index, calories, previous day minimum temperature and chronological time.

Among non-asthmatic children, FVI was inversely related to IL-8 but this association was not significant. FVI was significantly related to an increase of exhaled NO. No association with lung functions was observed.

### Association of MDI with IL-8 and lung functions

The associations between MDI and main outcomes for the asthmatic children are shown in Table 3. After accounting for air pollutants (O_3_, PM_2.5_) and confounding factors, including use of vitamin supplementation, MDI was not related to IL-8 or to exhaled NO or exhaled breath PH. However, it was significantly related to FEV_1 _and to FVC. A 1-point increase in MDI was associated with a 58 ml increase in FEV_1 _and a 75 ml increase in FVC.

**Figure 3:**
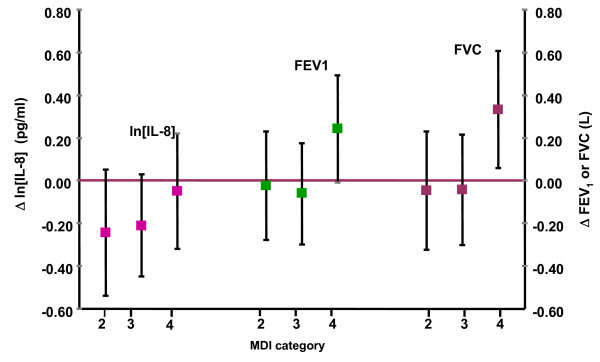
**Association between ln [IL-8] and lung function in asthmatic children living in Mexico City by categories of Mediterranean diet index (MDI) 2003-2005 [lnIL-8] model was adjusted for gender, body mass index, calories, 24-hr average O3 (pbb), previous day minimum temperature, corticoid use and chronological time**. Lung function models were adjusted for gender, body mass index, calories, 5-day accumulated moving average O3 (ppb), previous day minimum temperature and chronological time. Reference category 1.

When MDI was used as a categorical variable, we observed that FEV_1 _and FVC were significantly higher in the highest category when compare to the three lower categories (Figure 3). We calculate that children in the highest intake category of the MDI index (4) had a 15.3% higher FEV_1 _and a 16.5% higher FVC than children with the lowest category.

We observed a significant positive interaction between ozone level and MDI for FVC (p = 0.02) suggesting as for FVI, that the protective effect of MDI increased with increasing levels of ozone. When data were stratified by low (≤ 25 ppb, max 8-h moving average) and high ozone (≥ 38 ppb, max 8-h moving average) levels, we observed that the positive effect of MDI on FVC was significantly larger when exposure to ozone was in the highest quartile (Table 4).

Among non-asthmatic children, no significant association was observed between inflammatory markers or lung functions and MDI.

## Discussion

In this prospective cohort study, we observed that a higher intake of fruits and vegetables and close adherence to the MDI had a protective effect on the lung health of asthmatic children. This was observed over the range of 5-22 pulmonary function tests and repeated measurements of IL-8 in nasal lavage. We found a significant interaction between FVI and MDI and ozone exposure on IL-8 and lung functions, suggesting that high intake of fruits and vegetables and close adherence to the Mediterranean diet could modulate the adverse effect of O_3_.

Cross-sectional studies have shown that vitamin C and fruit intake are related to better lung function in adults [9,26]. Only one study has reported a similar effect in children [10]. Several studies have also suggested that specific foods might have an impact on asthma and allergies. Vegetables [9,27,28], fruits [9,27-29], dairy products [9,28,30,31] and fish [9,29,32,33] have been associated with reduced asthma risk in children, whereas fast food [34] and dietary fats [31] have been associated with an increased risk. Because foods can interact with one another, it has been suggested that dietary patterns derived from cluster or factor analysis [35] or the use of diet scores [35] are a useful approach for characterizing the diet of individuals and providing nutritional recommendations. Three recent studies have shown a positive impact of adherence to the Mediterranean diet on rhinitis [12] and asthma [13] in children. However, they were subject to bias as they relied on respiratory symptoms reported by parents, with no objective measurement of lung function or inflammatory response. In addition, none of these studies took into account exposure to ambient air pollution--a strong risk factor for respiratory health [36]. The present study, on the other hand, is a prospective study that evaluates the effect of dietary intake of fruits and vegetables and close adherence to the Mediterranean diet on inflammatory response and lung function among asthmatic and non-asthmatic children, taking exposure to air pollutants into account.

We used two types of indices, a fruit and vegetable index and a Mediterranean diet index. Our models were adjusted for total caloric intake as well as for potential confounding factors including gender, body mass index and the use of corticosteroids. While we observed an adverse effect of O_3 _and PM_2.5 _on inflammatory response and lung function as reported previously [7], the diet indices had a consistently beneficial effect on respiratory morbidity. Further adjustment for outdoor activity or distance to the child's residence to the closest high traffic road did not modify our results. A significant interaction was observed between FVI and ozone levels on IL-8 and lung function and between MDI and ozone levels on lung function.

High fruit and vegetable intake as defined by the FVI score appeared to be effective in reducing inflammation, as indicated by the lower IL-8 level in nasal lavage and in improving lung function. The high vitamin C, carotenoid and flavonoid content of the most frequently consumed fruits and vegetables (see *dietary intake*) might explain this effect, given the important role of antioxidants in protecting against endogenous and exogenous oxidative damage to the airways [37]. Other biologically active phytochemicals present in fruits and vegetables are also likely to have had a protective effect [26].

After accounting for air pollutants, close adherence to the Mediterranean diet was associated with higher lung function. Children in the highest MDI category had an FEV_1 _and FVC close to 16% higher than children in the lowest categories. The Mediterranean diet has been shown to have a high antioxidant capacity [11]. It is rich in hydrosoluble antioxidant vitamins and also in liposoluble vitamins and essential fatty acids such as vitamin E and omega-3 polyunsaturated fatty acid. These play a crucial role in protecting against the oxidant-induced lipoperoxidation of polyunsaturated fatty acids in cell membranes [38] and might also have an important role in modulating pulmonary response to oxidative stress. A relatively high consumption of fish (a source of omega-3 fatty acids), typical of the Mediterranean diet, combined with a low omega-6 intake from dietary fats is thought to modulate inflammation and immunological function, reducing the levels of proinflammatory mediators, such as tumor necrosis factor-alpha, which have been reported to be higher in asthmatic subjects [39].

An interaction of FVI and MDI and O_3 _exposure was observed on inflammatory response and lung function, suggesting that a diet rich in antioxidants and highly adherent to the Mediterranean diet could modulate the adverse effect of O_3 _on the respiratory health of asthmatic children. Ozone is a strong oxidant and high exposure can overwhelm antioxidant defenses and lead to decreased lung functions [7]. These results are in accordance with our recent findings on the modulating effect of vitamin C and Vitamin E supplementation on the adverse effect of O_3 _on lung function in asthmatic children [8] and suggest that protection against environmental insult can be achieved by an appropriate diet.

Adherence to the Mediterranean diet was assessed using an adaptation of the index developed by Trichopoulou [23] to evaluate population food habits, based on positive scoring for protective nutrients and negative scoring for detrimental nutrients. The index was modified slightly to fit a child population [40]. One of the advantages of this index is that it takes into account synergic effects or interactions between foods or nutrients [41], overrides correlations between different foods and minimizes error in the intake of specific nutrients, since it reflects the whole dietary pattern rather than specifically interesting nutrients or foods. In addition, use of the score improved statistical power, which is a concern when studying single nutrients or foods that account for small effects [40].

A number of issues need to be addressed in interpreting our results. Dietary intake was based on a food frequency questionnaire and the foods were then classified into 8 food groups to calculate the diet scores. This questionnaire had been validated in the Mexican population and the information on dietary intake was provided by the child's mother. Dietary intake is likely to vary with age among children; however, our study was focusing on acute and subacute effects and the time window covered by the questionnaire therefore appears adequate. While some error in reporting food intake is unavoidable, we believe it is random as both lung function tests and IL-8 measurements are objective outcomes and parents were unaware of the results. In addition, we obtained an average of 7 repeated measurements for lung function and 4 measurements for IL-8 per child, which increased our power, and we further adjusted for air pollution levels. We therefore believe our results provide a reliable estimation of the beneficial effect of diet on lung health in our population of asthmatic and non-asthmatic children.

Levels of IL-8 observed in our study were concordant with that observed in the nasal lavage in other studies [42-44]. We observed that the children with the highest intake (level 4) of FVI had a 13.7% lower IL-8 concentration than children in the first intake level.

We used repeated lung function measurements and learning curves could affect our

results; asthmatic children are used to performing spirometric tests and this would therefore more likely affect non-asthmatic children. However, excluding the first 2 spirometric tests from our analysis led to similar results.

The non-significant beneficial effect of diet observed among non-asthmatic children could be due to different factors. First, our sample size of non-asthmatic children was small because we had difficulties getting healthy children to visit our clinic. This would have influenced our power to detect a significant effect. Also, non-asthmatic children are known to have higher levels of antioxidants in the serum [45] and have less oxidative stress [46] It might therefore be easier to detect a beneficial effect among asthmatic children because of their susceptibility.

## Conclusions

Our findings indicate that a high dietary intake of commonly consumed fruits and vegetables and a close adherence to the Mediterranean diet had a protective effect on airway inflammation and lung function in our population of asthmatic children highly exposed to air pollutants. A "healthy diet" should be promoted to counteract environmental insults in asthmatic children. A stronger modulating effect was observed among asthmatic children exposed to high ozone levels.

## List of abbreviations

FVI: Fruit and vegetable index; MDI: Mediterranean diet index; FVC: Forced vital capacity; FEV_1_: Forced Expiratory Volume after 1 second; PM_2.5_: Fine particles; O_3_: Ozone; GINA: Global Initiative for Asthma; NO_2_: Nitrogen dioxide; RAMA: Government at four fixed-site central monitoring.

## Competing interests

The authors declare that they have no competing interests.

## Authors' contributions

IR: Developed the protocol, obtained funding for the project, and directed the data analysis and the writing of the manuscript. AB: participated in the protocol, data collection, standarization and realization of lung testing, interpretation and data analysis and writing of the manuscript. CE: data analysis and interpretation of the data; JT: participated on data collection; LH: participated on data analysis; DD: participated on measurement of IL-8 and pH and interpretation of the data; JDB: participated in the data collection and interpretation; and BD: participated in standardization of lung testing and data collection.

All authors have read and approved the final manuscript.
